# Optimization and Simultaneous Determination of Alkyl Phenol Ethoxylates and Brominated Flame Retardants in Water after SPE and Heptafluorobutyric Anhydride Derivatization followed by GC/MS

**DOI:** 10.1007/s10337-012-2293-6

**Published:** 2012-09-16

**Authors:** Tlou B. Chokwe, Jonathan O. Okonkwo, Linda L. Sibali, Esper J. Ncube

**Affiliations:** 1 Scientific Services, Rand Water, Vereeniging, 1930 South Africa; 2 Department of Environmental, Water and Earth Sciences, Tshwane University of Technology, Pretoria, 0001 South Africa; 3 Directorate of Research and Innovation, Tshwane University of Technology, Pretoria, 0001 South Africa

**Keywords:** GC–MS, Heptafluorobutyric anhydride derivatization, Simultaneous determination, Alkylphenol ethoxylates, Brominated flame retardants, Wastewater effluent and influent samples, Filtration

## Abstract

A gas chromatography–mass spectrometry (GC–MS) method was investigated for the simultaneous analysis of two types of endocrine disrupting compounds (EDCs), i.e., alkylphenol ethoxylates and brominated flame retardants (BFRs), by extraction and derivatization followed by GC–MS. Different solid phase extraction (SPE) cartridges (Cleanert PestiCarb, C_18_, Cleanert-SAX and Florosil), solvents (toluene, tetrahydrofuran, acetone, acetonitrile and ethyl acetate) and bases (NaHCO_3_, triethylamine and pyridine) were tested and the best chromatographic analysis was achieved by extraction with Strata-X (33 μm, Reverse Phase) cartridge and derivatization with heptafluorobutyric anhydride at 55 °C under Na_2_CO_3_ base in hexane. It was observed that APE together with lower substituted PBBs (PBB1, PBB10, PBB18 and PBB49), HBCD and TBBPA can be determined simultaneously under the same GC conditions. This simple and reliable analytical method was applied to determining trace amounts of these compounds from wastewater treatment plant samples. The recoveries of the target compounds from simulated water were above 60 %. The limit of detection ranged from 0.01 to 0.15 μg L^−1^ and the limit of quantification ranged from 0.05 to 0.66 μg L^−1^. There were no appreciable differences between filtered and unfiltered wastewater samples from Leeuwkil treatment plant although concentration of target analytes in filtered influent was slightly lower than the concentration of target analytes in unfiltered influent water. The concentrations of the target compounds from the wastewater treatment were determined from LOQ upwards.

## Introduction

A number of recent studies have indicated the widespread occurrence of several synthetic organic compounds in wastewater and as a result, significant research efforts have been devoted to their identification [1–4]. Among these compounds, nonylphenol (NP), nonylphenol ethoxylates (NPE), tetrabromobisphenol A (TBBPA), polybrominated diphenyl ethers (PBDEs), hexabromocyclododecane (HBCD) and polybrominated biphenyls (PBBs), present a significant research interest due to their extended use in several consumer and personal-care products and as flame retardants. Studies have shown that APEs and BFRs possess the ability to mimic natural hormones by interacting with the estrogen receptors [5–10]. Consequently, efforts have been made to determine their presence and concentrations in different environmental matrices including aquatic environment [1].

Many reports have been published on the determination of APEs and BFRs using various techniques. Some of the most frequently used methods for the analysis of these groups of compounds include: direct analysis using LC–MS [2], GC–ECD [9, 11], GC–FID [6], GC–MS [12], GC–MS/MS [13] and GC–HRMS [14, 15]. The use of gas chromatography for the detection of APEs is limited by the scarce volatility of the higher ethoxylated compounds [16–19]. On the other hand, the BFRs are volatile enough to be detected using GC, however, high BFRs congeners are very unstable and tend to decompose into lower congeners. Derivatization techniques may provide the answer to the simultaneous detection of these compounds in environmental matrices since derivatization has been used to volatilize non-volatile compounds and stabilize compounds that may undergo partial decomposition in the GC [19].

Several derivatization methods, such as acetylation, silylation and alkylation [16–22], have been used for the GC–MS analysis of phenolic compounds. Acetylation and methylation techniques are suitable for the analytes with high molecular weights. For the group of compounds covered in the present study, acetylation was used because of its quantitative reactions with various hydroxyl compounds at relatively moderate conditions. Among numerous acetylation reagents for derivatization of the hydroxyl group, 1-(trifluoroacetyl) imidazole (TFAI), heptafluorobutyric anhydride (HFBA) and pentafluoropropionic anhydride (PFPA) have widely been used [23]. The use of HFBA as a derivatizing agent for the determination of TBBPA in environmental samples has been reported [23], but never in the presence of APEs in wastewater samples.

**Fig. 1:**
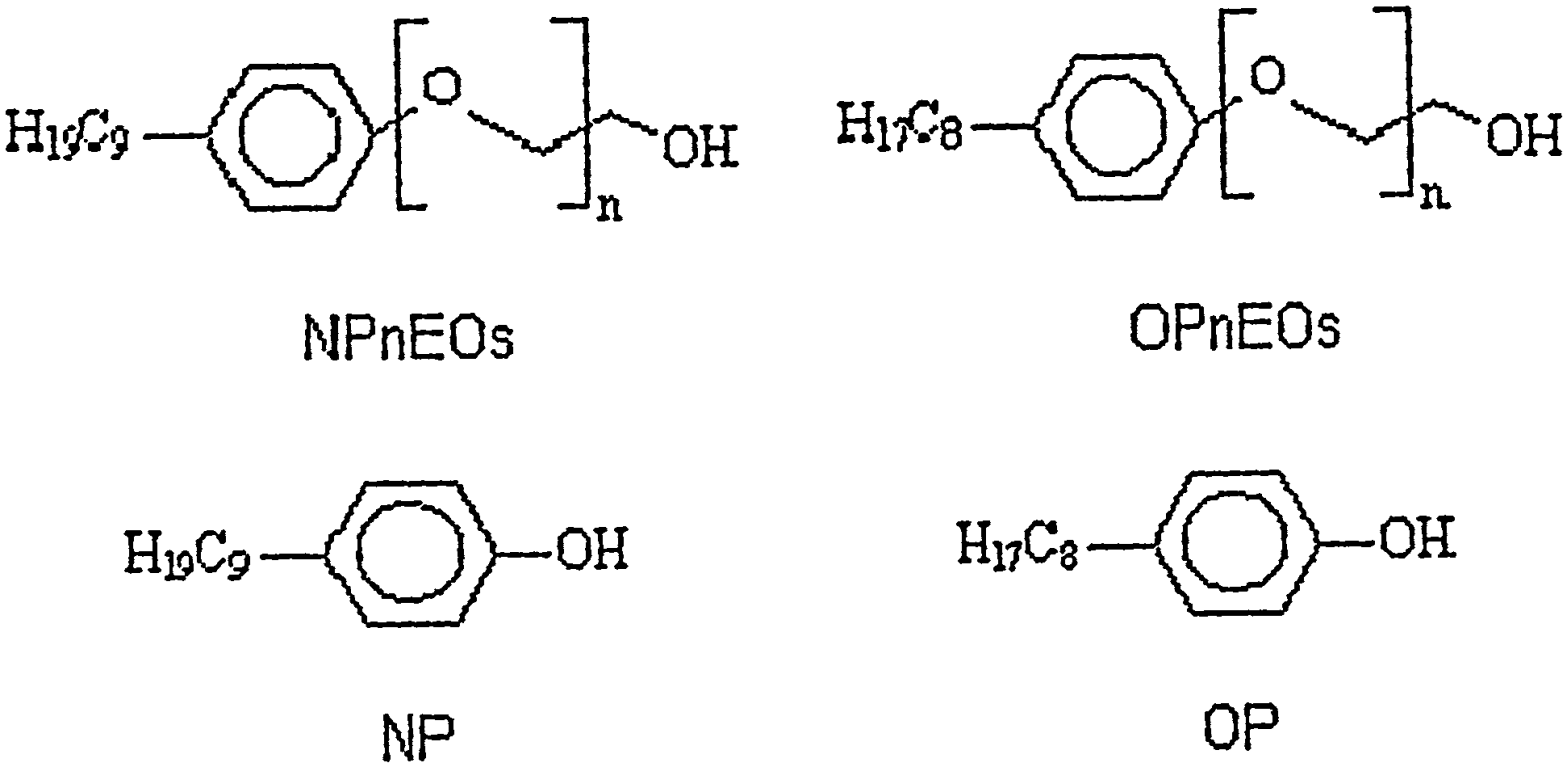
Structures of common APEs (nonyl- and octyl-phenol ethoxylates) and their metabolites

**Fig. 2:**
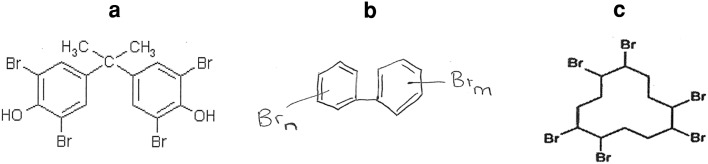
Structure of **a** TBBPA, **b** PBBs and **c** HBCD

This paper, therefore, reports on a simple and reliable procedure, based on SPE followed by HFBA derivatization and gas chromatography-mass spectrometry, for the simultaneous determination of APEs and BFRs in influent and effluent environmental samples obtained from a wastewater treatment plant.The approach adopted in the present study is seen to save analyses time and sample handling. Moreover, the impact of filtration of samples on the recoveries of these compounds in real samples was investigated (Figs. 1, 2).

## Experimental

### Standards and Reagents

Derivatizing agents, heptafluoro butyric anhydride (HFBA) and pentafluoro propionic anhydride (PFPA) were of analytical grade purchased from Sigma-Aldrich, South Africa. The solvents, acetone and hexane, used in the study were of GC grade and were used without further purification. The APEs and PBBs were purchased from Laboratories Dr. Ehrenstorfer-Schäfers, Augsburg, Germany. Only the NPE, NPPE and OPPE were of technical grade and the remaining APEs and PBBs were of analytical grade. Tetrabromo bisphenol A of technical grade as Firemaster BP4A and hexabromo cyclododecane of technical grade were purchased from AccuStandard, USA. Helium as He 5.5 pure was purchased from Air Product South Africa, Vereeniging.

## Method Development

### Derivatization Using HFBA

Into a vial, APs (1 mg L^−1^), APEs (4 mg L^−1^), PBBs (1 mg L^−1^), HBCD (2 mg L^−1^), and TBBPA (4 mg L^−1^), 1 mL hexane, 10.5 mg Na_2_CO_3_ and 75 μL HFBA were added and the content heated to 55 °C for 2 h and the derivatization was completed. Thereafter, the contents were cooled and the carbonate quenched with water. The organic phase was then drawn off and the volume made up to 1 mL with hexane. Thereafter, 1 μL was injected into the gas chromatography-mass spectroscopy for analysis.

### Instrumentation and GC–MS Conditions

An Agilent 6890 GC equipped with 5973 mass selective detector (MSD) was used for GC/MS analysis. The GC was equipped with a Gerstel autosampler. The injection port was fitted with a Cyclo Double Gooseneck 4 mm ID deactivated inlet liner (Restek, for Agilent GCs). The GC separation was initially conducted using 30 and 15 m DB5 with film thickness of 0.25 mm and internal diameter of 0.25 μm capillary column and later with Restek RTx-1614, capillary column (film thickness 0.10 μm, 15 m × 0.25 mm ID), (Chromspec cc South Africa). The GC–MS conditions used for analysis were as follows: carrier gas, He; linear velocity, 40 cm s^−1^; injector temperature, 275 °C; transfer line temperature, 300 °C; ion source, 150 °C. For analysis, 1 μL splitless injection was carried out by autosampler. The GC temperature program conditions were as follows: initial temperature 50 °C, heated to 120 °C by a temperature ramp of 7.5 °C/min then 275 °C by a temperature ramp of 15 °C/min then finally heated to 300 °C (held for 2 min) by a temperature ramp of 25 °C min^−1^.

### SPE and Derivatization of APEs and BFRs in MilliQ Water Sample

About 250 mL of MilliQ water acidified to pH 3 with acetic acid was spiked with 100 μl of standard, APs (1 mg L^−1^), APEs (4 mg L^−1^), PBBs (1 mg L^−1^), HBCD (2 mg L^−1^) and TBBPA (4 mg L^−1^), then extracted using SPE cartridge (Strata-X 33 μm polymeric reversed phase, 500 mg/6 mL). Before use, the SPE cartridge was conditioned with 6 mL of 30 % MeOH in DCM followed by the addition of 6 mL of MeOH at a flow rate of approximately 10 mL min^−1^. The sample was loaded into the conditioned cartridge by suction by means of a vacuum pump and, thereafter, the cartridge was dried for 1 h under vacuum. The compounds were eluted with 3 × 2 mL of mixture of DCM–hexane (4:1). The elutes collected were reduced to dryness under a gentle stream of nitrogen and then subjected to the derivatization and GC–MS analysis as described above.

### Limits of Detection and Quantification of Analytes

The instrument detection limit (IDL) was computed using the method described by Miller and Miller [24] given by the following equation:

1 $$ {\text{IDL }} = \, Y_{\text{b}} + 3S_{\text{b}} $$

where *Y*_b_ is the blank value and *S*_b_ is the standard error of the regression line. The noise and thresholds were set during column background run so as to eliminate noise spikes from being registered as peaks.

### Quantification of Analytes

The targeted analytes were quantified by peak area abundance using external standard method. A five point calibration curves were linear (*r* ^2^ = 0.98) across the concentration range of 0.2–1.0 μg L^−1^.

## Water Sample Collection and Extraction

### Wastewater Sample Collection

Environmental water samples were collected from the Leeuwkuil wastewater treatment plant located in the Vereeniging region, South Africa. Water samples were collected at the inlet (influent) and at outlet (effluent) using Winchester 250 mL brown bottle. The samples were acidified and placed in cooler bags, transported to the laboratory and stored in cold room set at a temperature of 4 °C. The samples were allowed to equilibrate at room temperature before use.

### Extraction and Derivatization of APEs and BFRs in Wastewater Sample

A total of 250 mL wastewater samples were collected from influent and effluent sources from a wastewater treatment plant. The influent samples were divided into filtered and unfiltered samples. The influent sample was first filtered and then spiked with APEs and BFRs standards, and spiked and then filtered. The filtration was carried out using Buchner flask fitted with a 0.47 μm pore size. The acidified wastewater samples were then subjected to the extraction and derivatization procedures as described earlier.

### Quality Control and Quality Assurance

The spiking method was used in the quality assurance process of analytical method due to unavailability of certified reference material for target compounds. Simulated water sample was spiked with 100 μL of standard mixture of 1.0 μg L^−1^ APs and PBBs; 2.0 μg L^−1^ HBCD and 4.0 μg L^−1^ APEs and TBBPA. The mixture was taken through the same extraction and derivatization procedure mentioned above prior to GC analysis. Quality assurance measures used in this study included running blanks with each sample set.

### Data Analysis

All samples were prepared in triplicate and from the triplicate measurement the mean concentrations were calculated and expressed as μg L^−1^. The mean and standard deviation were calculated from the measurements.

## Results and Discussions

### Derivatization

**Fig. 3:**
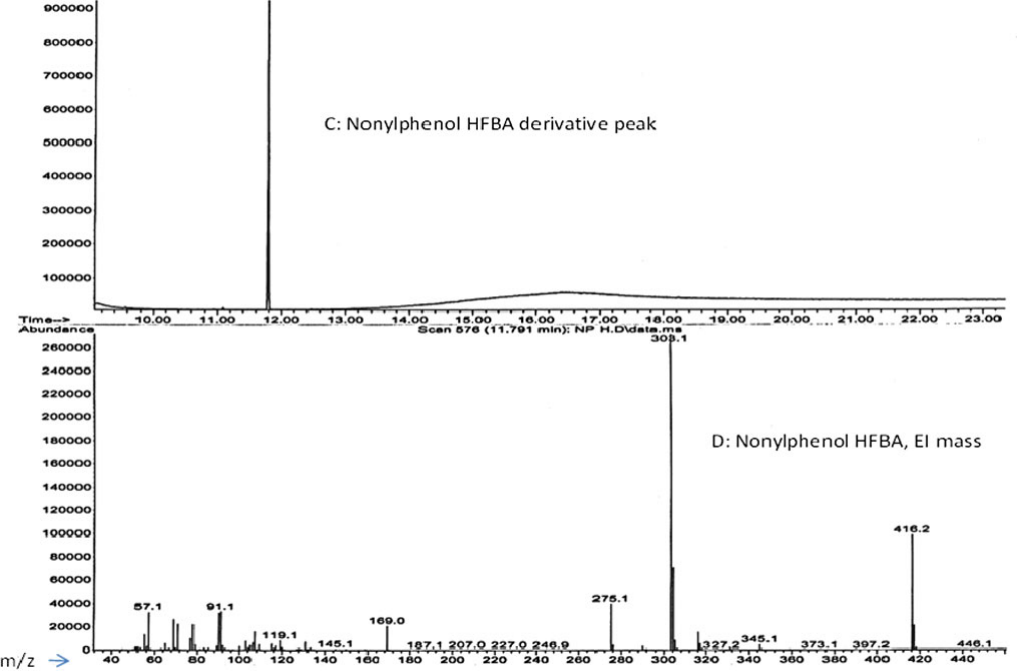
The *C* gas chromatogram and *D* EI-mass spectra of nonylphenol-HFBA derivative

In order to improve the selectivity and sensitivity of detection, phenolic compounds are often derivatized prior to GC–MS analysis [21]. Using nonylphenol as a benchmark for derivatization, different derivatization agents [1-(trifluoroacetyl) imidazole (TFAI), pentafluoropropionic anhydride (PFPA) and heptafluorobutyric anhydride (HFBA)], solvents (hexane, acetonitrile, toluene and acetone) and bases (NaHCO_3_, Na_2_CO_3_, pyridine and triethylamine) were compared in order to find optimal conditions for the derivatization of the targeted analytes. In the experiments conducted, it was observed that both the PFPA and HBFA in hexane with Na_2_CO_3_ as base provided stable derivatives, while the TFAI derivative was milky necessitating a further clean-up step. For further experiments, derivatization with HFBA was chosen because of quantitative reaction, the formation of stable products and availability of the agent. Figure 3 shows a nonylphenol HFBA derivative chromatogram obtained with a 30 m long DB5 column with EI-mass spectra.

**Fig. 4:**
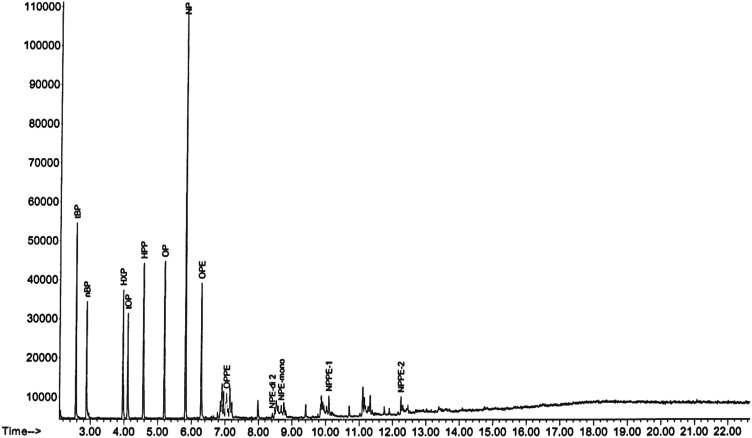
The GC chromatogram of derivatized APEs

The APEs where derivatized simultaneously using the optimized conditioned obtained from nonylphenol and the chromatogram is presented in Fig. 4. The change in retention time for nonylphenol was due to the use of a shorter column (15 m DB5). The change to a shorter column was to minimize the degradation of the products during analysis.

**Fig. 5:**
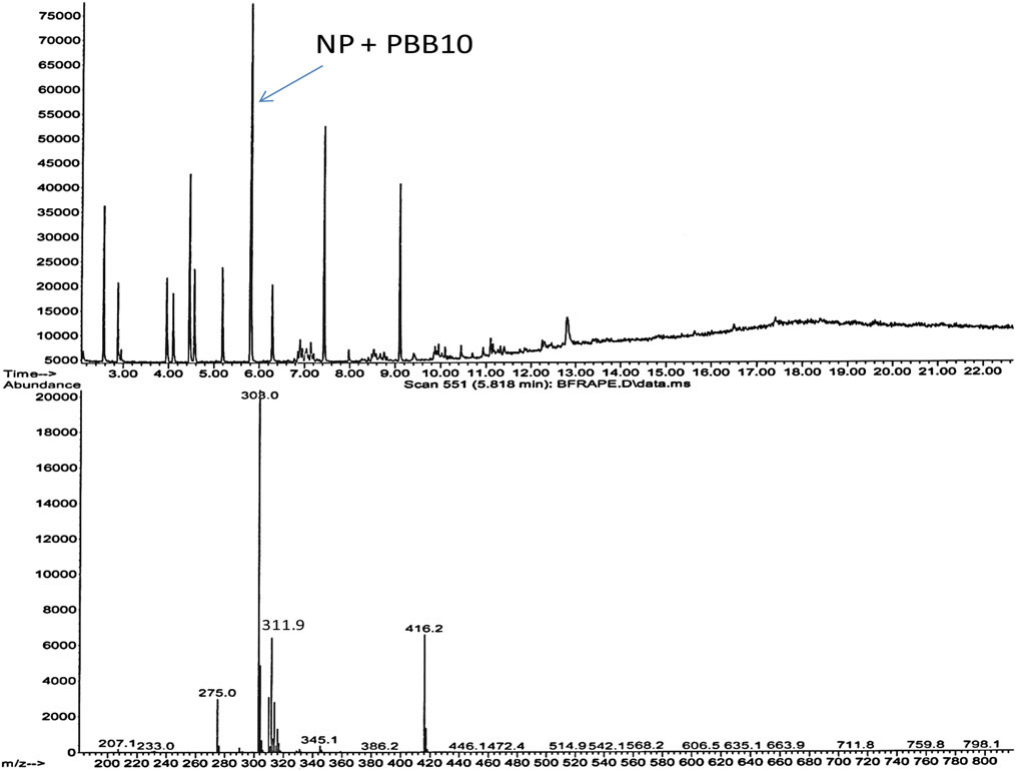
GC chromatogram and EI-mass spectra showing co-elution of PBB10 with NP

However, when the APs were analyzed together with PBBs (1, 10, 18 and 49), there was a co-elution of NP and PBB10 using a temperature ramp of 15 °C/min from 50 to 120 °C as shown in Fig. 5. As seen from Fig. 5, the sensitivity of PBB10 was low from the EI-mass fragmentation.

When this temperature ramp was reduced from 15 to 7.5 °C/min, the two compounds were separated. The ramp condition was, therefore, kept at 7.5 °C/min throughout the study. Due to low peak area abundance of PBBs from the EI-mass fragmentation, RTs 1614 column (15 m) was used in place of the DB5 (15 m) and was used further during the course of the analysis. The selected APEs and TBBPA were derivatized simultaneously in the presence of PBBs, (1, 10, 18 and 49) and HBCD as presented in Fig. 5.

**Fig. 6:**
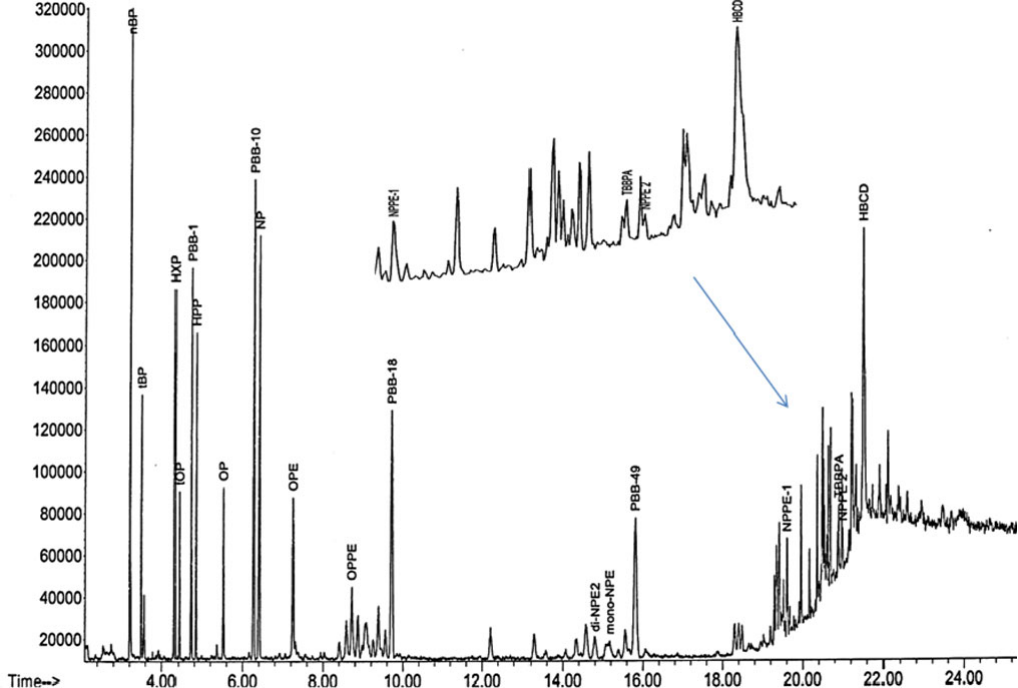
GC chromatogram of derivatized APEs and TBBPA in the presence of PBBs and HBCD

Although both the NPE and NPPE were used as technical mixtures, it was easy to identify their masses in the chromatograph (Fig. 6). It was observed that the NPE comprised nonylphenol ethoxylate (mono-NPE) and nonylphenol di-ethoxylate (di-NPE). The M^+^ ions used to identify the NPE, as shown in Table 1, were 433.2, 419 and 461 for mono-ethoxylate and 419, 475, 405, 433, and 504 for di-ethoxylate. There were also two sets of the penta-ethoxylate. The ions used to differentiate between the two penta-ethoxylates were 463, 477, 519 and 639 and the other was 551, 565, 607 and 639 and well separated (19.629 and 20.982 min). This additional fingerprint information may be very useful for the identification of these compounds in the complex matrix environmental samples. It was also observed that derivatization depended on the analyte structure, time and solvent. In this study, the derivatization reaction for the phenolic hydroxyl group was completed faster than that for the alcoholic hydroxyl groups. This phenomenon has been observed by Hoai et al. [18].

**Table 1:** Ions for selected ion monitoring of heptaflourobutyric derivatives

Analyte	Retention time (min)	Quantification ion (abundance)	Confirmation ions (abundance)
*t*-BP	3.202	331	346
n-BP	3.494	303	346
HXP	4.278	303	374.1
*t*-OP	4.481	331	
PBB-1	4.776	232	
HPP	4.824	303	388.1
OP	5.644	303	402
PBB-10	6.267	311.9	
NP	6.423	303	416.2
OPE	7.177	375	446
OPPE	8.858	389.1	375; 431; 361; 615
PBB-18	9.645	389.8	310.9; 232.0
PBB-49	15.802	469.7	390.8; 309.8
di-NPE2	14.636	419	433.1; 475; 405; 504
di-NPE1	15.803	433.2	419; 475; 405; 504
mono-NPE	15.190	433.2	419; 461
NPPE1	19.628	463.1	477.1; 519.3
TBBPA	20.848	726	
NPPE2	20.982	551	565, 607
HBCD	21.541	562.8	400.8; 319; 239

Solid phase extraction is the most widely used pre-concentration procedure. It is used not only to extract traces of organic compounds from environmental samples but also to remove interfering components from the matrix [1]. The performances of four different types of cartridges were initially examined using initially 1,000 mL spiked water (data shown for 4 cartridges, Table 2). Strata-X gave better recoveries for the extraction of APEs, while PestiCarb cartridges gave better recoveries for the extraction of BFRs compared to the other cartridges tested. Cai et al. [25] studied the effect of pH on extraction efficiencies of similar target analytes and the results showed extraction recovery (>95 %) for APEs (NP and *tert*-OP) remains relatively similar at pH 3–8, but recoveries of BPA were dramatically decreased to 60 % at pH above 8. Hoai et al. [18] reported that simultaneous determination of NPnEOs and their halogenated derivatives at pH 2–4 with HCl was found to be applicable for the extraction and elution of the analytes. On the basis of their experimental results, pH range of 3–4 was chosen as the pH of the sample solutions.

**Table 2:** Extraction results from four types of SPE cartridges

Compound	μg L^−1^					% Recovery			
	Exp. conc.	Strata-X	PestCarb	Florisil	C18	Strata-X	PestCarb	C18	Florisil
*t*-BP	0.40	0.06	0.01	0.00	0.00	15.00	2.50	0.00	0.00
n-BP	0.40	0.24	0.22	0.36	0.42	60.00	55.00	105.00	90.00
HXP	0.40	0.24	0.01	0.03	0.04	60.00	2.50	10.00	7.50
*t*-OP	0.40	0.23	0.02	0.05	0.05	57.50	5.00	12.50	12.50
PBB-1	0.20	0.01	0.15	0.00	0.00	5.00	75.00	0.00	0.00
HPP	0.40	0.29	0.02	0.06	0.16	72.50	5.00	40.00	15.00
OP	0.40	0.27	0.01	0.05	0.24	67.50	2.50	60.00	12.50
PBB-10	0.20	0.04	0.15	0.05	0.01	20.00	75.00	5.00	25.00
NP	0.40	0.19	0.01	0.08	0.25	47.50	2.50	62.50	20.00
OPE	0.40	0.43	0.34	0.42	0.42	107.50	85.00	105.00	105.00
OPPE	1.60	1.54	1.21	1.37	1.35	96.25	75.63	84.38	85.63
PBB-18	0.20	0.05	0.16	0.10	0.09	25.00	80.00	45.00	50.00
PBB-49	0.20	0.05	0.21	0.13	0.17	25.00	105.00	85.00	65.00
di-NPE2	1.60	1.09	1.02	1.02	1.45	68.13	63.75	90.63	63.75
di-NPE1	1.60	0.79	0.62	0.61	0.86	49.38	38.75	53.75	38.13
mono-NPE	1.60	1.14	0.88	0.97	1.20	71.25	55.00	75.00	60.63
NPPE1	1.60	0.29	0.36	0.25	0.39	18.13	22.50	24.38	15.63
TBBPA	1.60	1.52	0.18	0.23	0.53	95.00	11.25	33.13	14.38
NPPE2	1.60	0.21	0.19	0.25	0.16	13.13	11.88	10.00	15.63
HBCD	0.80	1.37	2.86	2.15	3.56	171.25	357.50	445.00	268.75

Cartridges conditioned with 5 mL ethyl acetate followed by 5 mL MeOH and 5 mL acidified water (pH 3 with HCl) and eluted with 3 × 2 mL ethyl acetate

Strata-X and PestiCarb cartridges were subjected to further test in order to select the cartridge with the better performance for all the analytes. It was observed that the Strata gave better extraction efficiency compared to PestiCarb when the former was conditioned with 30 % MeOH in DCM followed by MeOH and acidified water (acidified to pH 3 with acetic acid) and the extraction volume reduced from 1,000 to 250 mL.

Sibali et al. (2010) [6] confirmed APE loss by analytes retention in the sample bottle. In order to prevent the retention of analytes from the sample bottle, several volumes of methanol were added to the sample before the enrichment step to minimize this adsorption problem. As shown in Table 3, 1% methanol (2.5 mL MeOH in 250 mL sample) gave better results for most of the compounds. Taking into consideration the recoveries of all the analytes, 1% MeOH addition was chosen as the best condition.

**Table 3:** Effect of MeOH addition during SPE of APEs and BFRs

Compound	RT (min)	Exp. conc.	Conc. (μg L^−1^)			% Recovery		
			1 mL	2.5 mL	5 mL	1 mL	2.5 mL	5 mL
*t*-BP	3.202	0.400	0.27	0.33	0.25	67.50	82.50	62.50
n-BP	3.494	0.400	0.28	0.31	0.26	70.00	77.50	65.00
HXP	4.278	0.400	0.37	0.28	0.35	92.50	70.00	87.50
*t*-OP	4.481	0.400	0.34	0.25	0.32	85.00	62.50	80.00
PBB-1	4.776	0.400	0.11	0.26	0.07	27.50	65.00	17.50
HPP	4.824	0.400	0.28	0.21	0.28	70.00	52.50	70.00
OP	5.644	0.400	0.32	0.35	0.35	80.00	87.50	87.50
PBB-10	6.267	0.400	0.36	0.39	0.38	90.00	97.50	95.00
NP	6.423	0.400	0.24	0.27	0.27	60.00	67.50	67.50
OPE	7.177	0.400	0.53	0.40	0.59	132.50	100.00	147.50
OPPE	8.858	1.600	1.8	1.34	2.31	112.50	83.75	144.38
PBB-18	9.645	0.400	0.28	0.31	0.37	70.00	77.50	92.50
PBB-49	15.802	0.400	0.04	0.19	0.04	10.00	47.50	10.00
di-NPE2	14.636	1.600	3.03	2.05	4.49	189.38	128.13	280.63
di-NPE1	14.802	1.600	2.98	1.61	3.86	186.25	100.63	241.25
mono-NPE	15.190	1.600	2.87	1.64	0.95	179.38	102.50	59.38
NPPE1	19.629	1.600	3.21	3.85	2.56	200.63	240.63	160.00
TBBPA	20.848	1.600	1.02	1.23	1.31	63.75	76.88	81.88
NPPE2	20.982	1.600	3.12	1.26	1.06	195.00	78.75	66.25
HBCD	21.541	0.800	0.53	0.54	0.32	66.25	67.50	40.00

The results on the effect of washing the cartridge after extraction are tabulated in Table 4. After passing the sample through the cartridge, the cartridge was washed with appropriate amount of water (i.e., 5, 10 and 15 mL as indicated in Table 4) to remove any interference and then dried for 1 h. The results showed that washing of the cartridges had an overall negative effect on the recoveries of the compounds especially the brominated compounds as their recoveries were the most reduced with increased washing. With APEs, the effect was considered negligible. Based on these observations, cartridge washing step was not incorporated in the extraction of these analytes.

**Table 4:** The effect of washing cartridges after extraction step

Compound	RT (min)	Exp. conc.	Conc. (μg L^−1^)				% Recovery			
			0 mL	5 mL	10 mL	15 mL	0 mL	5 mL	10 mL	15 mL
*t*-BP	3.202	0.400	0.33	0.35	0.31	0.31	82.50	87.50	77.50	77.50
n-BP	3.494	0.400	0.36	0.36	0.33	0.33	90.00	90.00	82.50	82.50
HXP	4.278	0.400	0.36	0.36	0.4	0.35	90.00	90.00	100.00	87.50
*t*-OP	4.481	0.400	0.39	0.38	0.4	0.35	97.50	95.00	100.00	87.50
PBB-1	4.776	0.400	0.21	0.07	0.09	0.07	52.50	17.50	22.50	17.50
HPP	4.824	0.400	0.33	0.34	0.35	0.3	82.50	85.00	87.50	75.00
OP	5.644	0.400	0.33	0.35	0.35	0.31	82.50	87.50	87.50	77.50
PBB-10	6.267	0.400	0.53	0.3	0.51	0.36	132.50	75.00	127.50	90.00
NP	6.423	0.400	0.25	0.23	0.27	0.23	62.50	57.50	67.50	57.50
OPE	7.177	0.400	0.53	0.49	0.54	0.44	132.50	122.50	135.00	110.00
OPPE	8.858	1.600	1.72	1.56	1.85	1.57	107.50	97.50	115.63	98.13
PBB-18	9.645	0.400	0.34	0.27	0.34	0.3	85.00	67.50	85.00	75.00
PBB-49	15.802	0.400	0.24	0.03	0.04	0.03	60.00	7.50	10.00	7.50
di-NPE2	14.636	1.600	2.22	2.06	2.09	1.76	138.75	128.75	130.63	110.00
di-NPE1	14.802	1.600	2.09	2.26	3.16	2.25	130.63	141.25	197.50	140.63
mono-NPE	15.190	1.600	2.61	2.7	2.64	1.97	163.13	168.75	165.00	123.13
NPPE1	19.629	1.600	2.85	3.56	3.65	4.32	178.13	222.50	228.13	270.00
TBBPA	20.848	1.600	0.76	0.79	0.52	0.35	47.50	49.38	32.50	21.88
NPPE2	20.982	1.600	1.95	0.65	0.58	0.85	121.88	40.63	36.25	53.13
HBCD	21.541	0.800	0.58	0.22	0.3	0.41	72.50	27.50	37.50	51.25

*RT* retention time

Finally, triplicate extraction and derivatization of analytes were performed under the condition deemed optimum. The condition included the simultaneous derivatization of APEs in the presence of BFRs at 55 °C with HFBA, Na_2_CO_3_ with hexane as solvent for 2 h. The extraction included the conditioning of Strata-X cartridge with 6 mL of 30 % MeOH in DCM followed by addition of 6 mL of MeOH. The samples were acidified to pH 3 with acetic acid. As shown in Table 5, the percentage recoveries obtained ranged from 65 ± 7.07 % for PBB-1 to 167.82 ± 6.63 % for mono-NPE. Because of this high recovery and abundance of molecular ions during the development, the internal standard addition method was not used. Recoveries greater than 100 % of some analytes (i.e. PBB10, OPE, NPE and NPPE) may be because standards of technical grade were used instead of analytical grade which could not be sourced. With the exception of NP which exhibited a recovery of 66 %, all the target analytes in these study showed comparable recoveries with those reported [18, 21, 23, 25].

**Table 5:** Recoveries of APEs and BFRs in MilliQ water (*n* = 3)

Compound	RT (min)	Expected conc. (μg L^−1^)	Conc. (μg L^−1^)	Relative recovery (%)
*t*-BP	3.202	0.400	0.36 ± 0.035	90.00 ± 8.84
n-BP	3.494	0.400	0.36 ± 0.014	90.00 ± 1.41
HXP	4.278	0.400	0.39 ± 0.042	95.50 ± 10.61
*t*-OP	4.481	0.400	0.41 ± 0.028	102.00 ± 7.07
PBB-1	4.776	0.400	0.26 ± 0.028	65.00 ± 7.07
HPP	4.824	0.400	0.36 ± 0.035	90.00 ± 8.84
OP	5.644	0.400	0.36 ± 0.035	90.00 ± 8.84
PBB-10	6.267	0.400	0.56 ± 0.035	138.50 ± 9.19
NP	6.423	0.400	0.27 ± 0.021	66.25 ± 5.30
OPE	7.177	0.400	0.56 ± 0.042	140.00 ± 7.78
OPPE	8.858	1.600	1.75 ± 0.042	109.38 ± 2.65
PBB-18	9.645	0.400	0.33 ± 0.014	82.50 ± 3.53
PBB-49	15.802	0.400	0.27 ± 0.014	66.25 ± 5.30
di-NPE2	14.636	1.600	2.23 ± 0.014	139.38 ± 0.88
di-NPE1	14.802	1.600	2.13 ± 0.353	133.13 ± 20.33
mono-NPE	15.190	1.600	2.68 ± 0.071	167.82 ± 6.63
NPPE1	19.629	1.600	2.10 ± 0.071	131.25 ± 4.24
TBBPA	20.848	1.600	1.12 ± 0.007	69.69 ± 0.44
NPPE2	20.982	1.600	2.40 ± 1.704	110.32 ± 35.36
HBCD	21.541	0.800	0.61 ± 0.014	76.25 ± 2.83

### Limits of Detection and Quantification

In order to evaluate the experimentally found optimum conditions, linearity, limit of detection and quantification were determined. Linearity was checked by preparation of five different concentration levels from the APEs and BFRs standards. The relative standard deviations for the percentage recoveries as indicated in Table 5 were lower than 30 % except for NPPE-1 which was 35 %. This indicated a good repeatability of the extraction. The targeted analytes were quantified by peak area abundance using external standard method. A five point calibration curves were linear (*r* ^2^ = 0.98) across the concentration range of 0.2–1.0 μg L^−1^. As shown in Table 6, the calibration curves had good linear relationships using the standard solutions at five different concentration levels. The detection limits for the target analytes ranged from 0.01 (PBB-1) to 0.20 (di-NPE-2) at 95 % confidence level. Gatidou et al. [21] and Diaz et al. [22] found the LOD for NP, mono-NPE and di-NPE to be 0.02, 0.34 and 0.41 μg L^−1^, respectively, compared to LOD of 0.02, 0.15 and 0.30 μg L^−1^, respectively, obtained in this present study. These values (for NP, mono-NPE and di-NPE) were also comparable with the values obtained by Azevedo et al. [26].

**Table 6:** Quantitative calibration and detection limits of analytes

Compound	Range of standards (μg L^−1^)	Correlation co-efficient	LOD (μg L^−1^)	LOQ (μg L^−1^)
*t*-BP	0.2–1.0	0.992	0.03	0.08
n-BP	0.2–1.0	0.995	0.08	0.26
HXP	0.2–1.0	0.991	0.03	0.09
*t*-OP	0.2–1.0	0.994	0.02	0.07
PBB-1	0.2–1.0	0.997	0.01	0.05
HPP	0.2–1.0	0.995	0.02	0.07
OP	0.2–1.0	0.994	0.02	0.07
PBB-10	0.2–1.0	0.995	0.02	0.06
NP	0.2–1.0	0.992	0.02	0.08
OPE	0.2–1.0	0.993	0.02	0.08
OPPE	0.8–4.0	0.990	0.14	0.45
PBB-18	0.2–1.0	0.991	0.03	0.08
PBB-49	0.2–1.0	0.993	0.02	0.08
di-NPE2	0.8–4.0	0.993	0.20	0.66
di-NPE1	0.8–4.0	0.994	0.10	0.34
mono-NPE	0.8–4.0	0.990	0.15	0.51
NPPE1	0.8–4.0	0.997	0.07	0.23
TBBPA	0.8–4.0	0.992	0.10	0.34
NPPE2	0.8–4.0	0.986	0.13	0.42
HBCD	0.4–2.0	0.996	0.03	0.11

*LOD* limit of detection, *LOQ* limit of quantification

### Recoveries of APEs and BFRs in Wastewater Samples

In order to properly validate the method used in the present study, as well as the impact of filtration before and after spiking with standard test reagents, recovery experiments were performed on wastewater samples collected from a treatment plant. To accomplish this, extraction, derivatization and GC–MS procedures described earlier were repeated. The results are given in Table 7. The percentage recoveries in effluent ranged from 52.50 ± 2.13 to 168.75 ± 4.05 % while in the influent ranged from 32.50 ± 5.35 to 107.50 ± 3.53 %. When the influent water sample was filtered and spiked prior to SPE extraction and derivatization, it was observed that the recoveries of the target analytes improved as compared to when the influent was spiked and then filtered. The slight improvement in recoveries was attributed to the removal of particulate materials that may have retained the target analytes in the influent spiked and filtered sample. The effluent recoveries were comparable to the results reported [21].

**Table 7:** Recoveries of APEs and BFRs in wastewater

Corrected data compound	Expected conc.	Effluent spiked relative recovery (%)	Influent spiked filtered relative recovery (%)	Influent filtered spiked relative recovery (%)
*t*-BP	0.400	67.50 ± 2.02	52.50 ± 6.23	60.00 ± 3.33
n-BP	0.400	60.00 ± 3.00	50.00 ± 5.89	57.50 ± 8.13
HXP	0.400	70.00 ± 3.00	52.50 ± 9.23	60.00 ± 5.68
*t*-OP	0.400	52.50 ± 2.13	47.50 ± 12.36	55.00 ± 8.69
PBB-1	0.400	65.00 ± 9.36	60.00 ± 7.23	62.50 ± 13.21
HPP	0.400	62.50 ± 5.32	62.50 ± 1.23	60.00 ± 7.88
OP	0.400	72.50 ± 1.25	72.50 ± 15.23	65.00 ± 8.85
PBB-10	0.400	117.50 ± 9.19	77.50 ± 2.31	105.00 ± 1.23
NP	0.400	57.50 ± 3.50	67.50 ± 7.78	57.50 ± 5.35
OPE	0.400	82.50 ± 7.78	32.50 ± 5.35	90.00 ± 2.36
OPPE	1.600	57.50 ± 1.89	83.13 ± 6.23	102.50 ± 4.23
PBB-18	0.400	75.00 ± 5.33	55.00 ± 4.23	67.50 ± 3.98
PBB-49	0.400	62.50 ± 5.30	52.50 ± 6.23	70.00 ± 2.05
di-NPE2	1.600	75.00 ± 8.80	80.63 ± 18.23	96.25 ± 2.65
di-NPE1	1.600	80.00 ± 12.32	64.38 ± 9.98	102.50 ± 2.50
mono-NPE	1.600	56.88 ± 6.63	64.38 ± 3.68	107.50 ± 3.53
NPPE1	1.600	168.75 ± 4.05	67.50 ± 4.68	74.38 ± 4.39
TBBPA	1.600	72.50 ± 2.65	58.13 ± 17.32	52.50 ± 1.39
NPPE2	1.600	53.75 ± 15.23	89.38 ± 3.69	76.88 ± 2.97
HBCD	0.800	56.25 ± 8.32	57.50 ± 7.23	72.50 ± 8.63

*Effluent spiked* final water leaving the plant spiked, *influent spiked filtered* raw water entering the plant spiked then filtered, *influent filtered spiked* raw water filtered then spiked

### Levels of APEs and BFRs in the Environmental Samples

**Fig. 7:**
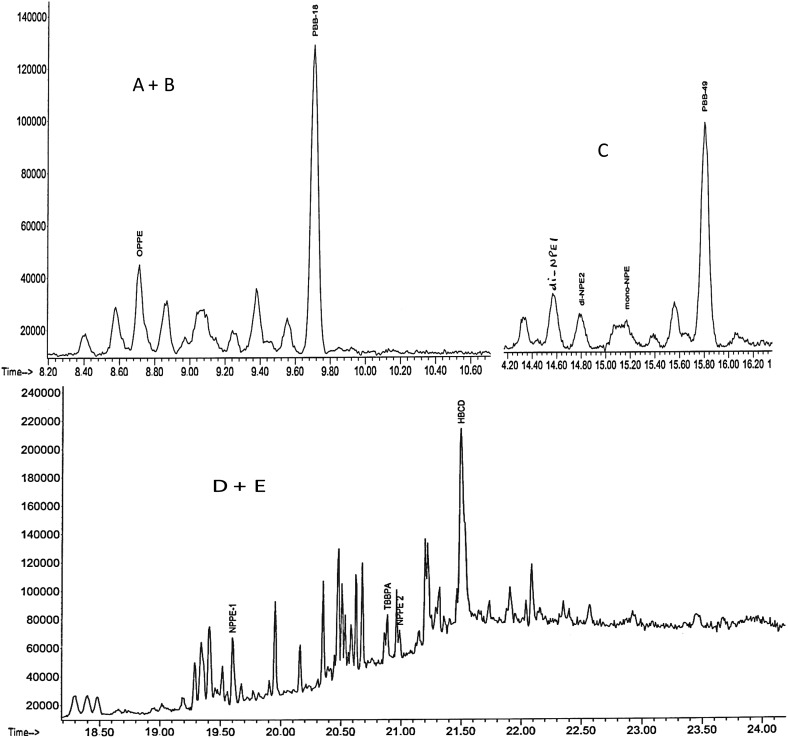
Expanded peaks of (*A*+*B*) OPPE and PBB18; (*C*) di-NPE(1+2), PBB49; and (*D*+*E*) NPPE-1, TBBPA, NPPE-2 and HBCD

The developed method was successfully applied to wastewater samples taken from the wastewater treatment plant in order to determine the concentrations of the target compounds in the water samples. The chromatograms of the analytes are shown in Fig. 7, and their concentrations in Table 8. From Fig. 7, the peaks were fairly separated. As seen in Table 8, most of the target compounds determined in the samples were obtained at levels lower than those found in other studies [21] except for di-NPE1, mono-NPE, NPPE1 and NPPE2 with concentrations ranging from 10.268 to 10.615 μg L^−1^, 3.014 to 16.373 μg L^−1^, 5.553 to 15.156 μg L^−1^ and 13.449 to 21.971 μg L^−1^, respectively, in both filtered and unfiltered samples. However, as observed by Sibali et al. [6], there were no appreciable differences between filtered and unfiltered wastewater samples from Leeuwkuil treatment plant although concentration of target analytes in filtered influent water was slightly lower than the concentration of target analytes in unfiltered influent raw water. Also the concentrations of the analytes in the effluent were generally lower than the concentrations in filtered and unfiltered influent samples.

**Table 8:** Concentrations of APEs and BFRs in wastewater samples

Corrected data compound	Effluent (μg L^−1^)	Influent (μg L^−1^)	Influent raw (μg L^−1^)
*t*-BP	ND	ND	0.095
n-BP	ND	ND	ND
HXP	ND	ND	ND
*t*-OP	ND	ND	0.105
PBB-1	ND	ND	ND
HPP	ND	ND	ND
OP	ND	ND	ND
PBB-10	ND	ND	ND
NP	ND	ND	ND
OPE	ND	ND	0.092
OPPE	1.461	4.566	4.259
PBB-18	ND	ND	ND
PBB-49	ND	ND	ND
di-NPE2	ND	ND	6.474
di-NPE1	0.550	10.615	10.268
mono-NPE	2.092	3.014	16.373
NPPE1	0.972	5.553	15.156
TBBPA	3.269	6.629	6.806
NPPE2	3.126	21.971	13.449
HBCD	0.142	0.1400	0.139

*Effluent* final water leaving the plant, *influent* raw water filtered then acidified and MeOH added, *influent raw* raw water acidified and MeOH added then filtered, *ND* not detected

## Conclusions

A simultaneous SPE extraction and derivatization followed by GC–MS method was developed for the determination of selected APEs and BFRs in wastewater treatment sample. The derivatization procedure involved the reaction of these compounds, simultaneously in the presence of lower congeners of PBBs and HBCD, with HFBA under Na_2_CO_3_ base at 55 °C for 2 h. SPE extraction and GC–MS analysis of the derivatized compounds gave sharp peaks with good and high sensitivity for the analytes. The GC–MS analysis was completed in less than 22 min. The results of this study demonstrated that the represented method showed acceptable relative recoveries for the determination of APEs and BFRs in wastewater samples.

The developed method showed good recoveries of 65 ± 7.07–167.82 ± 6.63 % for the target compounds and adequate LOD and LOQ that ranged from 0.01 to 0.20 μg L^−1^ and 0.05 to 0.66 μg L^−1^, respectively. When the conditions developed were optimized and applied to environmental wastewater samples, the analytes were detected at low levels with the exception of nonylphenol penta-ethoxylates (NPPE2) which gave inexplicable high concentration value. The presented method had shorter analysis time and was simple.
